# In Cervical Myelopathy: Clinical Effect of the Posterior Myelon Shifting After Dorsal Decompression and Instrumentation

**DOI:** 10.3390/jcm14124319

**Published:** 2025-06-17

**Authors:** Yazan Noufal, Marcus Richter, Philipp Hartung, Felix Schmitz, Matthias Fröhlich, Philipp Drees, Yama Afghanyar, Martin Naisan

**Affiliations:** 1 Spine Center, St. Josefs Hospital Wiesbaden, 65189 Wiesbaden, Germany; mrichter@joho.de (M.R.); phartung@joho.de (P.H.); fschmitz@joho.de (F.S.); mnaisan@joho.de (M.N.); 2 Department of Orthopaedic Surgery, Trauma Surgery and Sports Traumatology, University of Witten/Herdecke, Cologne-Merheim Medical Centre (CMMC), 51109 Cologne, Germany; froehlichm@kliniken-koeln.de; 3 Department of Orthopaedics and Traumatology, University Medical Center of the Johannes Gutenberg University Mainz, 55131 Mainz, Germany; philipp.drees@unimedizin-mainz.de (P.D.); yama.afghanyar@unimedizin-mainz.de (Y.A.)

**Keywords:** cervical spondylotic myelopathy, cervical spine, spine surgery, Japanese Orthopaedic Association, spinal cord shift, C5-palsy

## Abstract

**Objectives:** Cervical spondylotic myelopathy (CSM) is a progressive neurological disorder caused by spinal cord compression in the cervical spine. The Japanese Orthopaedic Association (JOA) score is commonly used to quantify neurological impairment. Surgical decompression is the main treatment, aiming to relieve pressure on the spinal cord and improve neurological function. Historically, the primary goal was to halt disease progression; however, recent studies suggest an improvement even in cases with mild symptoms. The extent to which the shifting of the spinal cord contributes to clinical improvement remains controversial. **Methods:** This study included 95 patients who underwent dorsal decompression and instrumentation for cervical myelopathy between 2020–2024. Patients were followed up at 3 and 12 months post surgery. **Results:** Dorsal decompression resulted in a significant improvement in neurological function as measured by the JOA score at three months and one-year post-operation (0.0004 and 0.006, respectively). The average posterior spinal cord shift (PSS) was 3 mm. The prevalence of C5-palsy was 3.1%. Linear and logistic multivariable regression showed no significant relationship between PSS and JOA score alteration or subjective clinical improvement, but a multivariable regression analysis identified the ASA score as a significant negative predictor of neurological improvement. **Conclusions:** Dorsal decompression in CSM is a safe procedure concerning neurological complications and does not only stop the worsening of symptoms but indeed improves the JOA score and the subjective clinical situation. Although PSS was observed postoperatively, the extent of PSS showed no statistically significant relationship with JOA score improvement.

## 1. Introduction

Cervical spondylotic myelopathy is a progressive neurological condition caused by compression of the spinal cord at the cervical level. The most common causes include degenerative changes such as cervical spondylosis; ossification of the posterior longitudinal ligament (OPLL); and disc herniation as well as instabilities, like in the case of rheumatological diseases [1,2]. It is the leading cause of spinal cord dysfunction in adults and is associated with a wide range of symptoms that include motor weakness, sensory deficits, gait instability, and impaired fine motor skills [3,4]. In advanced stages, it can lead to significant neurological impairment and a severe decline in quality of life. Early diagnosis and timely intervention are therefore critical to preventing irreversible damage and improving functional outcomes [5].

Surgical decompression is widely recognized as the most effective treatment for moderate to severe cervical myelopathy [6]. Among the available surgical techniques, posterior approaches such as laminectomy with or without fusion are commonly employed, particularly in cases involving multilevel stenosis or ventrodorsal compression [7,8,9]. These procedures aim to relieve pressure on the spinal cord by expanding the spinal canal and reducing mechanical stress. A key mechanism contributing to clinical recovery after posterior decompression is the posterior shifting of the spinal cord (PSS) [10,11]. This term refers to the measurable posterior shifting of the spinal cord observed on postoperative MRI imaging. This shift not only alleviates direct compression but also promotes improved vascular perfusion and facilitates neural recovery [12]. Understanding the extent of the PSS and its relationship to clinical outcomes has become an area of growing interest in recent years [13,14].

A feared complication is the C5 palsy, a form of neurologic deficit of the C5-nerve that is thought to be caused by PSS after dorsal decompression of the segment C4/5 [15].

The Japanese Orthopaedic Association (JOA) score is one of the most widely used tools for assessing neurological function in patients with cervical myelopathy before and after surgery [16]. This scoring system evaluates motor function in both the upper and lower extremities, sensory function, and bladder control, providing a comprehensive measure of disease severity. Improvements in JOA scores following posterior decompression have been well-documented in the literature, with many studies demonstrating significant recovery in neurological function postoperatively [17]. However, patient outcomes can vary considerably depending on factors such as age, preoperative symptom severity, duration of symptoms, and underlying pathology. Additionally, complications such as postoperative kyphosis or insufficient decompression may limit the effectiveness of these procedures in certain cases.

Specifically, this study seeks to address three key questions: (1) To what extent does posterior decompression facilitate PSS? (2) How does the PSS correlate with improvements in neurological function as measured by JOA scores? (3) Which patient- and procedure-specific factors influence the outcome?

## 2. Methods and Data

The study was conducted according to the guidelines of the Declaration of Helsinki and approved by the ethics committee Ethikkommission Landesärztekammer Hessen, application number 2025-3982-evBO on 21 February 2025. As this study is retrospective in nature, a need for informed consent was waived by the ethics committee.

### 2.1. Study Population

We collected data from 95 patients of our clinic from 1 January 2020 to 31 December 2024, who underwent dorsal decompression and instrumentation in cases of cervical myelopathy. The data was extracted from the intrahospital information system (ORBIS, Agfa Healthcare GmbH, Bonn, Germany).

Patients were eligible for inclusion if they were above 18 years of age. Acute compression of the spinal cord by trauma, tumor, or bleeding was an exclusion criterion.

We recorded the demographic data of the patients, the preoperative JOA score, the clinical symptoms, other neurologic or bone-related diseases, the narrowed segments (see Table 1), the instability of the vertebral bodies, the type of spinal canal stenosis, and previous surgeries of the cervical spine. Details of the collected parameters are provided in Table S1 (Supplementary Materials).

In total, 22 patients have undergone ventral decompression and stabilization before, one patient has already had a dorsal decompression of the cervical spine but not related to the myelopathy, and 5 patients have undergone dorsal instrumentation of the cervical spine.

### 2.2. Study Design

After surgery, patients have been evaluated for intraoperative and postoperative complications, JOA Score, and subjective neurological improvement of the clinical situation at discharge. Furthermore, the postoperative MRI scans were assessed for PSS (see Section 2.3).

Patients have been summoned to follow-up 3 months and 12 months after surgery.

**Figure 1:**
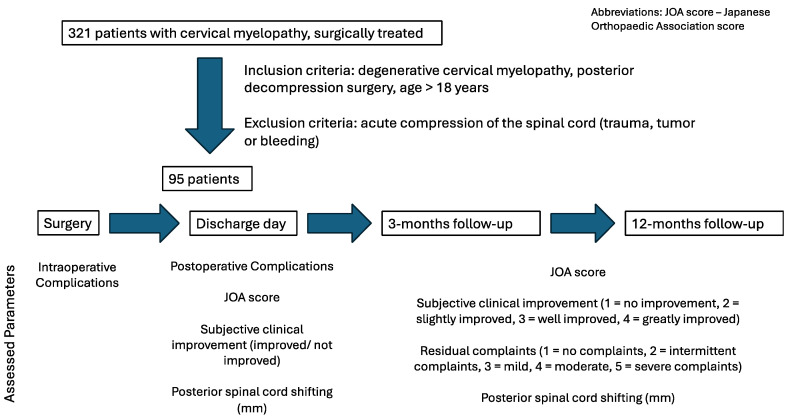
Study design flow chart. The diagram illustrates the study flow and outcome assessment schedule for patients undergoing posterior decompression surgery due to degenerative cervical myelopathy. Out of an initial cohort of 321 patients surgically treated for cervical myelopathy, 95 patients fulfilled the inclusion criteria and were included in the analysis. Inclusion criteria comprised age over 18 years and posterior decompression surgery for degenerative cervical myelopathy. Patients with acute spinal cord compression due to trauma, tumor, or hemorrhage were excluded. Postoperative follow-up assessments were conducted at discharge, 3 months, and 12 months after surgery. At each follow-up time point, both clinical and radiological parameters were recorded. Clinical parameters included intraoperative and postoperative complications, the Japanese Orthopaedic Association (JOA) score, and subjective clinical improvement categorized as “improved” or “not improved” at discharge. At 3 and 12 months, subjective clinical improvement was graded on a 4-point scale (1 = no improvement, 2 = slightly improved, 3 = well improved, and 4 = greatly improved). Residual complaints were also assessed using a 5-point scale (1 = no complaints, 2 = intermittent complaints, 3 = mild, 4 = moderate, and 5 = severe complaints). Radiologically, the posterior shifting of the spinal cord (in millimeters) was measured via postoperative MRI at the corresponding time points. Abbreviation: JOA score—Japanese Orthopaedic Association score.

The JOA Score, subjective clinical improvement, and residual complaints were assessed as well as the PSS (see Figure 1).

In total, 27 patients received an MRI before discharge, 30 patients at 3-month-follow-up, and 17 patients at 12-month-follow-up. In total, 9 patients did not attend the 3-month-follow-up and 17 patients did not attend the 12-month-follow-up.

The JOA score was recorded at discharge in 72 patients, at 3-month-follow-up in 47 patients, and at 12-month-follow-up in 60 patients. In 16 cases, the 12-month-follow-up was still pending.

Of the patient collective, there are 47 patients in which the actual JOA scores were mentioned in the follow-up reports and concurrently have carried out postoperative MRI of the cervical spine. In 69 patients, both postoperative MRI data and information on subjective clinical improvement and residual complaints were available.

### 2.3. Outcome Measures

The primary outcome of this study was the improvement of neurological function after surgical decompression, which was assessed using the Japanese Orthopaedic Association (JOA) score. The JOA score evaluates motor and sensory function in the upper and lower extremities as well as bladder function. It was recorded at discharge, at 3-month follow-up, and at 12-month follow-up, with scores available for 72, 47, and 60 patients, respectively.

In addition to the JOA score, subjective clinical improvement and residual complaints were extracted from standardized follow-up reports. At discharge, patients were categorized into “improved” and “non-improved”. At 3-month and 12-month follow-up, improvement was rated on a four-point scale: 1 = no improvement, 2 = slightly improved, 3 = well improved, and 4 = greatly improved. Residual complaints were rated on a five-point scale: 1 = no complaints, 2 = intermittent complaints, 3 = mild complaints, 4 = moderate complaints, and 5 = severe complaints.

As a secondary outcome, the extent of postoperative dorsal shifting of the spinal cord was measured using a 1.5 Tesla scanner (Siemens Healthineers, Erlangen, Germany). T2-weighted sagittal and axial sequences were acquired in all cases.

**Figure 2:**
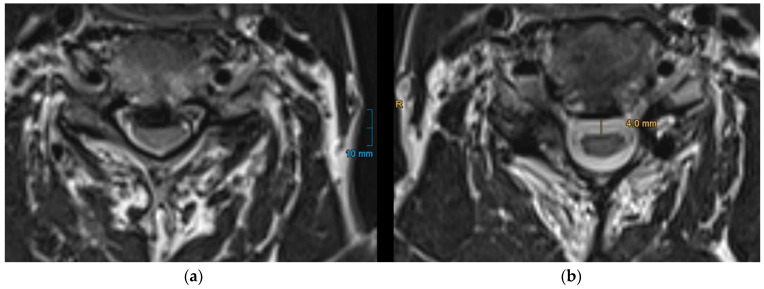
Measurement of posterior spinal cord shifting (PSS) on axial T2-weighted MRI. PSS is defined as the distance from the approximated anterior limitation of the spinal cord to the anterior spinal canal. (**a**): preoperative MRI showing the spinal cord compression before surgery; (**b**): postoperative MRI illustrating the degree of PSS.

PSS was quantified by measuring the perpendicular distance from the approximated anterior limitation of the spinal cord to the anterior spinal canal (Figure 2) as it is already described in previous studies [15,18]. For patients with multilevel stenosis, this measurement was performed at each narrowed segment, and the mean PSS was calculated. To ensure measurement reliability, two independent observers (a senior and a junior spine surgeon) performed the measurements for all patients. Inter-rater reliability was assessed using intraclass correlation coefficient (ICC). The resulting ICC value was 0.91 (95% CI 0.86–0.95), indicating excellent agreement between raters.

### 2.4. Surgical Procedure

All patients underwent posterior cervical decompression via laminectomy under general anesthesia. The procedures were performed with the patient in a prone position using a Mayfield head fixation system. After a midline incision and subperiosteal dissection, the laminae of the affected cervical segments were resected to achieve adequate spinal cord decompression. In all cases, posterior stabilization was achieved using either lateral mass screws or pedicle screws. The choice of implant depended on the patient’s cervical anatomy. The construct was completed with rods, and alignment was verified intraoperatively. Wound closure was performed in layers over suction drains.

### 2.5. Statistical Analysis

The present assessments were performed using computer-assisted analyses. The necessary programming was performed in the R language using the R software package available on the Comprehensive R Archive Network (CRAN) at https://cran.r-project.org and Microsoft Excel. The distribution of data was examined using the Shapiro–Wilk test for normality. As the data did not follow a normal distribution, we applied a non-parametric test, the Wilcoxon test. In addition, we used a linear and logistic multivariable regression. Statistical significance was defined as *p* < 0.05.

## 3. Results

### 3.1. Improvement of the Neurological Outcome

Our study demonstrated a progressive improvement in Japanese Orthopaedic Association (JOA) scores following surgery. At discharge, in the total patient collective it showed an increase of 1.3 points (±1.4), or 11% (*p* = 0.07), which was only a positive tendency. However, at three months, the mean JOA score significantly improved to 14.31 (±2.15), representing a 1.64-point increase (15.6%, *p* = 0.0004). Details of the collected parameters are provided in Table S1 (Supplementary Materials).

This improvement was maintained at one year, with a mean score of 14.25 (±2.48) and a 1.67-point increase (16.7%, *p* = 0.006), indicating a sustained positive surgical outcome. Notably, this change approaches or exceeds the minimal clinically important difference (MCID) for the JOA score, which has been reported to range between 1.5 and 2.0 points. Therefore, the observed improvement can be considered to be not only statistically significant but also clinically meaningful.

Linear regression analysis reveals a statistically significant negative relationship between the level of the preoperative JOA score and its improvement after surgery. Specifically, for every one-unit increase in preoperative JOA, the model predicts a decrease of 0.04496 units in improvement (*p* < 0.001, R-squared = 0.2774).

Subjective clinical improvement was available in 69 patients. At 3-month follow-up, 87% of patients reported a subjective improvement in their neurological status, with 38% describing their condition as “greatly improved” (score 4), 26% as “well improved” (score 3), and 23% as “slightly improved” (score 2). Only 13% of patients reported no improvement (score 1), and no patients reported a worsening of symptoms.

Regarding residual complaints, most patients reported mild or no symptoms at follow-up. Specifically, 28% of patients indicated no residual complaints (score 1), 26% reported intermittent symptoms (score 2), 30% had mild persistent symptoms (score 3), and 16% reported moderate to severe residual complaints (scores 4–5). These findings reflect an overall favorable subjective outcome following posterior decompression surgery.

### 3.2. Posteior Shifting of the Spinal Cord

PSS occurred by an average of 3.004 mm (±1.8 mm) after the surgical intervention. This displacement showed no correlation with the number or specific levels of narrowed segments in the cervical spine. 

### 3.3. Factors Influencing the Neurological Outcome

In multivariable linear regression analysis, a higher ASA score was significantly associated with reduced improvement in the JOA score at 12 months (β = –1.99, *p* = 0.014). PSS showed a positive trend toward greater JOA improvement (β = 0.38, *p* = 0.094), as did BMI (β = 0.14, *p* = 0.094), although neither reached statistical significance. Other variables, including age, preoperative JOA score, neurologic comorbidities, prior surgery of the cervical spine, bone-related diseases, spinal instability, and intra- or postoperative complications, showed no significant association with JOA improvement.

In the logistic regression model assessing subjective clinical improvement (defined as a score ≥3 on the 4-point scale), none of the examined factors—including PSS, age, ASA, BMI, preoperative JOA, or intra- and postoperative complications—were significantly associated with better patient-reported outcomes. However, operative complications and higher ASA scores were again associated with reduced odds of subjective improvement (OR = 0.26 and OR = 0.51, respectively), indicating a possible negative trend.

A full overview of both regression models, including coefficients, odds ratios, confidence intervals, and *p*-values, is presented in Table 2.

**Figure 3:**
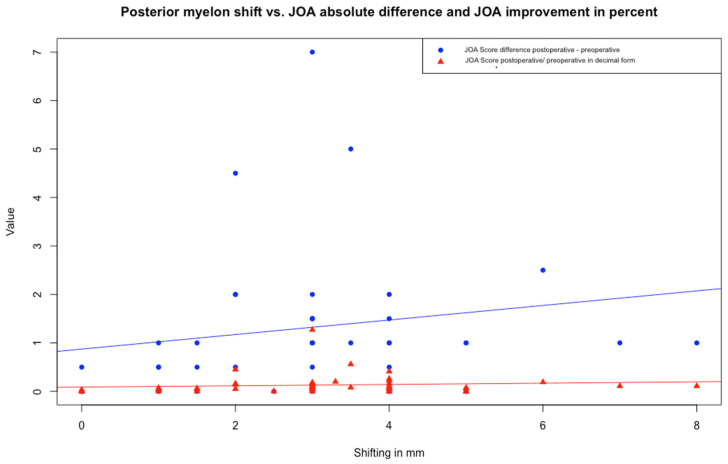
Scatter plot with linear regression analysis illustrating the relationship between posterior spinal cord shifting (PSS) and improvement of the JOA score as difference (postoperative–preoperative) and improvement in decimal form (postoperative/preoperative). Each dot represents a patient (in blue the JOA score difference, in red the improvement in decimal form). Regression lines illustrate the direction. Both models showed low R-squared values (3.08% and 1.33%, respectively). Abbreviation: JOA Score—Japanese Orthopaedic Association Score.

Linear regression analysis revealed no significant relationship between PSS and either JOA score difference or quotient. Both models showed low R-squared values (3.08% and 1.33%, respectively) (Figure 3).

**Figure 4:**
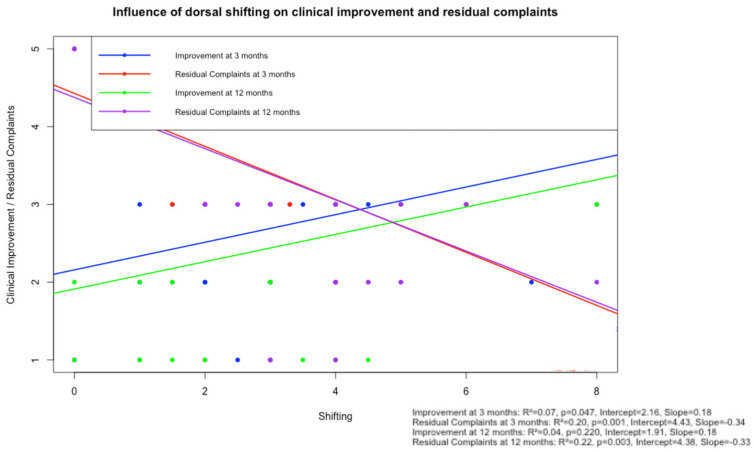
Scatter plot with linear regression analysis illustrating the relationship between posterior spinal cord shifting (PSS) and subjective clinical improvement and residual complaints, assessed at 3-month and 12-month follow-up. Each dot represents a patient (blue: subjective clinical improvement at 3 months; green: subjective clinical improvement at 12 months; red: residual complaints at 3 months; and purple: residual complaints at 12 months). Regression lines illustrate the direction. All models showed low R-squared values (0.04–0.22). Abbreviation: JOA Score—Japanese Orthopaedic Association Score.

In a separate linear regression model (Figure 4), PSS showed a weak, non-significant correlation with subjective improvement at 12 months (R^2^ = 0.04, *p* = 0.220, intercept = 1.91). These findings are consistent with the results of the multivariable logistic regression, in which no significant predictive value of PSS on dichotomized subjective improvement was observed (*p* = 0.49).

Interestingly, the number of narrowed segments and the level of stenosis did not affect JOA score improvement.

Of the 95 patients included in the study, 92 (96.8%) presented with concentric spinal canal stenosis, while ventral and dorsal compressions were observed in only two patients and one patient, respectively. Due to this distribution, no statistical subgroup analysis based on the type of compression was conducted.

### 3.4. Incidence of C5-Palsy

There were 3 cases of C5-palsy out of our 95 cases. In all three cases, the segment C4/5 had been decompressed. In total, 57 patients had the segment C4/5 operated on; thus, in about 5.3% of those patients C5-palsy occurred.

The first patient developed severe paresis of the deltoid and biceps muscles (1/5) on postoperative day 7. Two revision surgeries with re-decompression of C4/5 were performed. The motor deficit improved to 3/5 shortly after revision, and full recovery was documented at 12-month follow-up.

The second patient developed a mild deltoid paresis (4/5) on postoperative day 1, which was treated with high-dose corticosteroids, leading to full recovery within the follow-up period.

The third patient also developed C5 palsy on postoperative day 1, with a deltoid and biceps paresis graded 3/5. Corticosteroid pulse therapy was initiated, resulting in partial recovery to 4/5 motor strength.

## 4. Discussion

The incidence of hospitalizations related to cervical spondylotic myelopathy (CSM) is estimated at 4.04 per 100,000 person-years, with higher rates observed in older individuals and males. Additionally, the number of patients undergoing surgical treatment for CSM has significantly increased in recent years [19].

The surgical treatment consists of a spinal decompression with or without instrumentation of the vertebrae bodies [20]. The surgical approach is from either ventral, dorsal, or combined ventrodorsal. The ideal approach is the subject of discussion. Papavero et al. published a tool (7-letter code) to determine the most adequate approach [21]. In general, in a multisegmental concentric cervical stenosis a posterior approach with laminectomy is advised. The decompression of the spinal cord is attained indirectly by PSS. The incidence of C5 palsy is reported to be higher following posterior decompression procedures [15]. The exact mechanism remains unclear, but it is hypothesized to be related to the PSS. At our center, this approach is frequently employed. This study aimed to evaluate the effect of distraction on clinical outcomes.

The surgical treatment of CSM has consistently demonstrated significant and sustained improvements in neurological function across numerous studies. Our findings align with this trend, showing a progressive enhancement in Japanese Orthopaedic Association (JOA) scores following laminectomy surgery. While the improvement was not significant at discharge (1.3 points, *p* = 0.07), it became significant at three months (1.64 points, *p* = 0.0004) and was maintained at one year (1.67 points, *p* = 0.006). These results are consistent with the broader literature on CSM surgical outcomes [22].

A key aspect of our study was the examination of PSS following surgery, which averaged 3.004 mm (±1.8 mm).

The multivariable regression analysis revealed that a higher ASA score was significantly associated with reduced improvement in the JOA score at one-year follow-up. This finding aligns with the clinical expectation that patients with greater systemic comorbidities tend to have poorer postoperative functional recovery.

Interestingly, operative complications showed a strong negative effect on both objective (JOA) and subjective outcomes, although this association did not reach statistical significance. This likely reflects a limited number of complication events in our cohort and potential underpowering of the model to detect subtle but clinically relevant effects. Nonetheless, this trend warrants further investigation in larger cohorts.

As anticipated, the preoperative JOA score was inversely related to postoperative improvement, reflecting a ceiling effect: patients with milder baseline impairment had less room for measurable recovery.

Regarding the posterior spinal cord shift (PSS), the results were inconclusive. A non-significant positive trend was observed in the linear model, while the logistic model showed an inverse but also non-significant association. This finding aligns with the work of Nori et al., who reported that PSS did not affect the recovery rate of JOA scores. Their study concluded that PSS is not mandatory for satisfactory functional recovery, a notion supported by our results [23].

However, the relationship between PSS and functional outcomes remains a topic of debate in the literature. Chen et al., for instance, found a significant correlation between the recovery rate and PSS in their univariate analysis [24]. This discrepancy highlights the complex nature of CSM and the potential influence of various factors on surgical outcomes.

While all patients underwent laminectomy with posterior instrumentation, minor technical variations may have influenced the degree of PSS. The lack of a significant correlation between PSS and clinical outcomes might be influenced by several potential confounding factors. These include variations in the degree and chronicity of spinal cord compression or individual recovery capacity. Furthermore, the retrospective nature of the study limits control over surgical nuances and postoperative rehabilitation, which may also modulate outcomes. Future prospective studies with standardized protocols and stratified subgroup analyses are needed to better delineate these influences.

## 5. Limitations

This study is subject to important methodological limitations that must be clearly acknowledged. As a retrospective, single-center study, it is inherently prone to selection bias and limited control over confounding variables. Furthermore, the single-center setting restricts the external validity of the findings; as a result, it may not be generalizable to other institutions or patient populations. Of the 95 patients originally enrolled, only 47 had complete datasets available for the analysis of the primary outcome. This incomplete data set introduces a considerable risk of selection bias, as the included subgroup may not fully represent the overall patient population. Additionally, this may compromise the statistical power of this study, limiting the ability to detect subtle but potentially meaningful associations or differences. As a result, some effects may remain undetected, and the generalizability of our findings may be limited.

Moreover, relying solely on the JOA score to quantify clinical improvement carries its own limitations: it is an ordinal scale prone to ceiling effects in milder cases, focuses primarily on motor and sensory domains while overlooking patient-reported disability and quality of life, and may suffer from inter-rater variability in subjective items such as gait and hand function. To provide a more comprehensive assessment of postoperative outcome, future investigations should consider supplementing the JOA score with additional standardized tools—such as the Neck Disability Index, visual analog pain scales, or generic quality-of-life measures—which together can better capture the full spectrum of functional recovery and patient experience.

Prospective studies and randomized controlled trials may provide more definitive insights into risk factors and causal relationships.

## 6. Conclusions

Our findings have potential implications for surgical planning in CSM. Laminectomy indeed significantly improves the patient’s outcome. The lack of correlation between PSS and functional improvement challenges the notion that greater cord decompression necessarily leads to better outcomes. The incidence of C5-palsy is low, and therefore the posterior decompression seems to be a safe procedure regarding postoperative neurological complications.

## Figures and Tables

**Table 1:** Demographic data of the patient collective (mean values +/− standard deviation; except for the ASA status, the median value is given) and overview of neurological diseases, bone-related diseases, and stenosed segments (in number of patients and percentages). Abbreviations: JOA—Japanese Orthopaedic Association Score, ASA—American Society of Anesthesiologists.

**General Characteristics**	**Mean Value +/− Standard Deviation**	**Neurologic Comorbidities**	**Number (and Percentages)**
Age (years)	70.5 +/− 9.8	Carpal tunnel syndrome	8 (8.4%)
Body weight (kg)	77.8 +/− 18.3	Apoplex	3 (3.2%)
Height (in m)	1.71 +/− 0.09	Encephalopathy	1 (1.1%)
Body mass index (kg/ m^2^)	26.5 +/− 5.6	Mengioma	1 (1.1%)
Preoperative JOA Score	12.5 +/− 2.38	M. Parkinson	1 (1.1%)
Number of stenosed segments	2.2 +/− 0.98	Polyneuropathy	10 (10.5%)
ASA status (1 to 4)	3 (median value)	Spinal canal stenosis thoracic or lumbar spine	14 (14.7%)
		Tremor	1 (1.1%)
		Restless legs syndrome	1 (1.1%)
**Bone-Related Comorbidities**	**Number (and Percentages)**	**Levels of Cervical Stenosis**	**Number (and Percentages)**
Klippel Feil syndrome	4 (4.2%)	C1/2	2 (2.1%)
OPLL	5 (5.3%)	C2/3	6 (6.3%)
Rheumatoid Arthritis	11 (11.6%)	C3/4	43 (45.3%)
M. Bechterew	1 (1.1%)	C4/5	57 (60%)
DISH	1 (1.1%)	C5/6	57 (60%)
		C6/7	41 (43.2%)
		C7/Th1	6 (6.3%)

**Table 2:** Multivariable regression analysis of predictors for JOA improvement (linear model) and subjective clinical improvement (logistic model). Abbreviations: ΔJOA—improvement in Japanese Orthopaedic Association score at 1-year follow-up; OR—odds ratio; CI—confidence interval; ASA—American Society of Anesthesiologists classification; BMI—body mass index; and PSS—posterior spinal cord shift.

Model	Variable	Coefficient	Odds Ratio	95% CI (Coef.)	95% CI (OR)	*p*-Value
Linear (ΔJOA)	Intercept	1.98	-	−8.29 to 12.25	-	0.684
	PSS	0.38	-	−0.08 to 0.84	-	0.094
	Age (years)	0.03	-	−0.06 to 0.11	-	0.484
	ASA (1–4)	−1.99	-	−3.51 to 0.47	-	0.014
	BMI (kg/m2)	0.14	-	−0.03 to 0.31	-	0.094
	Preoperative JOA score	−0.18	-	−0.45 to 0.09	-	0.172
	Neurologic disorders	−0.41	-	−2.05 to 1.22	-	0.593
	Prior cervical spine surgery	−0.40	-	−2.62 to 1.82	-	0.702
	Bone-related disease	−0.66	-	−2.47 to 1.14	-	0.441
	Segmental instability	−0.42	-	−1.89 to 1.05	-	0.551
	Operative complications	−2.31	-	−6.51 to 1.9	-	0.257
Logistic (Subjective Clinical Improvement)	Intercept	−1.31	0.27	−3.17 to 0.56	0.04 to 1.75	0.173
	PSS	−0.25	0.78	−0.97 to 0.46	0.38 to 1.59	0.494
	Age (years)	0.06	1.06	−0.02 to 0.14	0.98 to 1.15	0.136
	ASA (1–4)	−0.67	0.51	−1.95 to 0.62	0.14 to 1.86	0.308
	BMI (kg/m^2^)	−0.01	0.99	−0.13 to 0.10	0.88 to 1.10	0.846
	Preoperative JOA score	−0.01	0.99	−0.24 to 0.21	0.79 to 1.23	0.903
	Neurologic disorders	0.30	1.35	−0.96 to 1.56	0.38 to 4.77	0.641
	Prior cervical spine surgery	−0.78	0.46	−2.22 to 0.66	0.11 to 1.93	0.284
	Bone-related disease	0.29	1.34	−1.01 to 1.60	0.36 to 4.95	0.658
	Segmental instability	0.01	1.01	−1.06 to 1.08	0.35 to 2.95	0.984
	Operative complications	−1.35	0.26	−3.21 to 0.50	0.04 to 1.65	0.151

## Data Availability

All data analyzed during this study are included in the Supplementary Information files.

## Supplementary Materials

The following supporting information can be downloaded at: https://www.mdpi.com/article/10.3390/jcm14124319/s1: Table S1: Complete dataset containing all clinical, demographic and radiological parameters collected and analyzed in this study. Each row corresponds to a single patient record, and columns represent the respective variables.

## Abbreviations

OPLL	ossification of the posterior longitudinal ligament
PSS	posterior spinal cord shift
JOA	Japanese Orthopaedic Association
ASA	American Society of Anesthesiologists
BMI	body mass index
CSM	cervical spondylotic myelopathy
DISH	Diffuse Idiopathic Skeletal Hyperostosis
